# Poor Glycemic Control in East Africa: Prevalence, Risk Factors and Public Health Implications in Diabetes Management

**DOI:** 10.1002/edm2.70233

**Published:** 2026-05-04

**Authors:** Fanny Eseohe Onohuean, Mary Onohuean, Haron Olot, Hope Onohuean

**Affiliations:** ^1^ Department of Nursing and Midwifery, Faculty of Health Science Metropolitan International University Kisorot Uganda; ^2^ Biomolecules, Metagenomics, Endocrine & Tropical Disease Research Group (BMETDREG) Kampala International University Ishaka‐Bushenyi Uganda; ^3^ Biopharmaceutics Unit, Department of Pharmacology & Toxicology, School of Pharmacy Kampala International University Ishaka‐Bushenyi Uganda

**Keywords:** diabetes mellitus, East Africa, epidemiology, glycemic control, public health, risk factors

## Abstract

**Background:**

Diabetes mellitus remains a major public health concern in East Africa, and poor glycaemic control continues to drive avoidable complications, deaths and pressure on already stretched health systems.

**Objective:**

To estimate the prevalence of poor glycemic control and describe the main factors associated with it among people living with diabetes in East Africa.

**Methods:**

This review synthesized evidence from observational studies, cross‐sectional surveys and regional health databases identified through PubMed, Scopus and Web of Science, following PRISMA guidance. Sociodemographic, clinical and behavioural indicators were examined to identify common patterns and predictors of poor glycaemic control. The review also considered how measurement approaches shaped reported estimates.

**Results:**

Fifty records were identified across PubMed (10), Scopus (23) and Web of Science (17). After screening, 37 records were eligible for full‐text review, and 15 studies met the inclusion criteria for evidence synthesis. Across the region, poor glycemic control was consistently high, ranging from 60% to 85%. Most studies were facility‐based and cross‐sectional. Glycemic control was assessed mainly using HbA1c, commonly defined as ≥ 7% or > 7.5%, and less frequently by fasting blood glucose, typically ≥ 7.2 mmol/L or > 130 mg/dL. Type 2 diabetes was the dominant population studied, with fewer mixed cohorts and only one study focused on type 1 diabetes. Factors repeatedly linked to poor control included older age, longer duration of diabetes, poor medication adherence, limited access to care, low health literacy, inadequate diabetes education, insulin use, comorbidities, diabetic complications, unhealthy diet, physical inactivity, sedentary behaviour, substance use and limited self‐management support.

**Conclusion:**

Poor glycemic control is alarmingly common among people with diabetes in East Africa and reflects intertwined clinical, behavioural and health‐system challenges. Region‐specific strategies are needed to strengthen primary care, improve diabetes education, expand affordable monitoring and treatment and enhance surveillance to guide policy and resource allocation.

## Graphical abstract

In East Africa, poor glycaemic control is common (about 60%–85%), usually defined by HbA1c ≥ 7% (or > 7.5%) and sometimes fasting glucose. Older age, longer duration, comorbidities and barriers to adherence and access drive the burden—supporting tiered, access‐focused interventions.

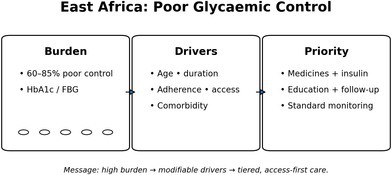

## 1 Background

Diabetes mellitus comprises a heterogeneous set of disorders, but the contemporary burden in East Africa is driven predominantly by type 2 diabetes (T2D), with type 1 diabetes (T1D) contributing a smaller–yet clinically important–share through earlier onset, lifelong insulin dependence and disproportionate risk of acute complications. In practice, health‐system planning must therefore address both (i) high‐volume T2D care in primary care and (ii) reliable insulin access, education and monitoring for T1D, particularly in children and adolescents [1, 2].

The global prevalence of diabetes has risen dramatically in recent decades, with the International Diabetes Federation reporting that approximately 537 million adults (20–79 years) were living with diabetes in 2021, and this number is projected to increase to 643 million by 2030 and 783 million by 2045 [3]. Various studies have suggested that the number of affected people will rise from 422 million to 642 million worldwide by 2040 [3, 4]. Over the past decades, East Africa has witnessed a concerning rise in diabetes mellitus, aligning with regional and global epidemiological trends. Rapid urbanization, changes in dietary patterns and reduced physical activity have contributed significantly to this shift [5]. Within this evolving landscape, poor glycemic control—often quantified via elevated glycated haemoglobin (HbA1c) or fasting blood glucose (FBG)—substantially burdens patients and health systems alike. However, effective glycemic control is essential in managing diabetes and preventing its complications [6]. Glycemic control is typically monitored using HbA1c, a blood test that reflects average blood glucose levels over the past two to three months [7]. An HbA1c level of 7% (53 mmol/mol) or lower is often recommended for most adults with diabetes to minimize the risk of complications [8]. Poor glycemic control can lead to long‐term complications and significantly impact the quality of life of the affected person [7, 9], leading to considerable health complications, including cardiovascular Disease [9], heart attack and stroke [10].

Recent studies highlight alarmingly high prevalence rates of poor glycemic control across East African nations [11, 12, 13, 14, 15]. Central to this epidemiological challenge are recurring demographic and clinical correlates such as advanced age, low educational attainment, extended duration of diabetes, insulin‐only treatment regimens and presence of comorbidities [12, 16, 17]. Behavioural determinants—such as poor dietary adherence, inadequate physical activity, khat chewing, alcohol use and limited use of self‐monitoring tools—further stratify risk [11, 13, 18]. Moreover, systemic barriers are evident in this region. Widespread deficiencies in self‐management support, uneven access to medications and monitoring devices and limited diabetes education undermine glycemic control, particularly in resource‐constrained and rural settings [17, 19]. Genetic factors unique to sub‐Saharan populations—such as variants affecting HbA1c reliability—raise concerns about adequate screening and monitoring [5]. This milieu underscores an urgent need for a comprehensive epidemiological prevalence of poor glycemic control and its characteristic features in the East African region to inform the stakeholders and research community engagement. This meta‐synthesis of the available evidence in the region will provide a step towards actualizing standardized measures and addressing behaviour, system and biological dimensions associated with poor glycemic control. Furthermore, diabetes care is often constrained by limited primary care capacity, uneven access to essential medicines and poor glycaemic monitoring, highlighting the need for tailored interventions that strengthen diabetes management across the region.

## 2 Methodology

### 2.1 Review Design and Reporting

This study is a systematic review with narrative evidence synthesis (no meta‐analysis), reported in line with PRISMA (Preferred Reporting Items for Systematic Reviews and Meta‐Analyses) 2020 guidance. A completed PRISMA checklist and the study selection flow diagram are provided as Data S1. The systematic review evaluated the epidemiological incidence of poor glycaemic control and its defining characteristics among patients with diabetes mellitus in East Africa. The research complied with PRISMA standards [20, 21, 22].

### 2.2 Data Sources and Search Strategy

Systematic searches were conducted in electronic databases such as PubMed, Scopus and Web of Science for pertinent papers published from January 2015 to August 2025. Search criteria encompassed combinations of ‘diabetes mellitus’, ‘glycaemic control’, ‘HbA1c’, ‘prevalence’, ‘risk factors’, and ‘East Africa’, country‐specific terms such as Ethiopia, Kenya, Uganda, Tanzania and Rwanda.

### 2.3 Inclusion and Exclusion Criteria

Studies were included if they: (a) were conducted in East African countries; (b) reported a quantitative estimate of glycaemic control (HbA1c and/or FBG) with a prespecified threshold for poor control; and (c) examined at least one related factor grouped a priori as demographic/socioeconomic (e.g., age, sex, education, income), clinical (e.g., duration of diabetes, therapy type, comorbidities/complications), behavioural/lifestyle (e.g., diet, physical activity, substance use, adherence), or health‐system/access (e.g., affordability/availability of medicines, monitoring, clinic access, education/support). Studies were included if they: (1) were conducted in East African countries; (2) reported on glycaemic control among patients with type 1 or T2D; (3) defined poor glycaemic control using HbA1c > 7% or FBG > 130 mg/dL; and (4) were published in English. Studies without definitive glycaemic thresholds and reviews and editorials were removed.

### 2.4 Data Extraction and Quality Assessment

Two independent reviewers gathered information about the study's features, prevalence of poor glycaemic control and related factors. Discrepancies were resolved by consensus. The Joanna Briggs Institute critical assessment checklist for prevalence studies was used to rate the quality of the included papers.

### 2.5 Data Analysis and Presentation

A narrative synthesis was employed to delineate the study's features, prevalence of poor glycaemic control, essential demographic factors, and clinical characteristics linked to poor glycaemic management.

## 3 Results

**FIGURE 1:**
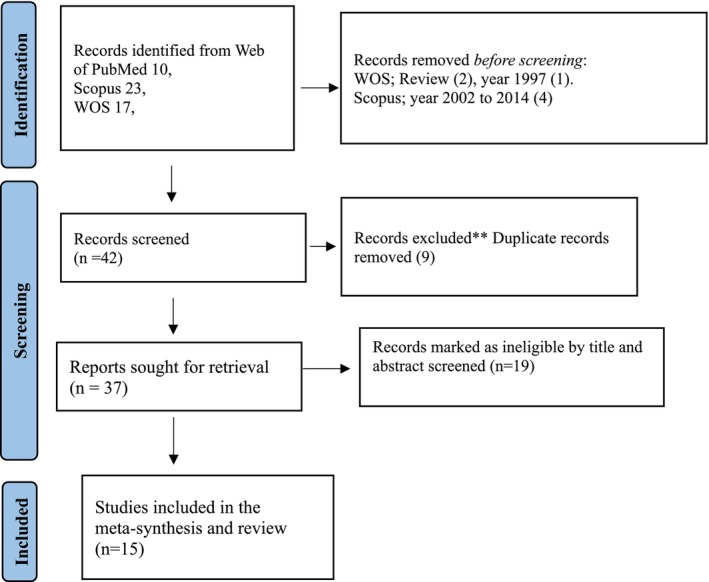
Flowchart of PRISMA guideline for the study selection for the meta‐synthesis and review.

A total of 50 articles were retrieved from three electronic databases: PubMed (*n* = 10; 20%), Scopus (*n* = 23; 46%) and Web of Science (*n* = 17; 34%). During the initial screening phase, seven articles (14%) were excluded for not meeting the predefined inclusion criteria, including two review articles and five studies conducted outside the target publication years (see Data S1). Data normalization and de‐duplication were performed using ScientoPy and the fBasics R package [20, 22], identifying and removing nine duplicate records (18%). After screening titles, abstracts and full texts for relevance to the research objective—specifically studies reporting poor glycaemic control (defined as HbA1c > 7% or FBG > 130 mg/dL) and associated demographic, epidemiological, clinical, or behavioural risk factors—19 articles (38%) were excluded. Ultimately, 37 full‐text articles (66%) were assessed for eligibility, and 15 studies met all inclusion criteria and were included in the narrative evidence synthesis, as illustrated in the (PRISMA flow diagram; Data S1 and Figure 1).

### 3.1 Prevalence of Poor Glycemic Control Among Diabetes Patients in the East African Region

Our findings on meta‐synthesis of published studies conducted between 2015 and August 2025 across five East African countries—Ethiopia, Kenya, Uganda, Tanzania and Rwanda—reveal consistently high rates of poor glycemic control among individuals living with diabetes mellitus Table 1. Across the included studies, most evidence was facility‐based and cross‐sectional. Glycaemic control was assessed mainly with HbA1c (typically ≥ 7% or > 7.5%) and, less often, FBG (≥ 7.2 mmol/L or > 130 mg/dL). T2D predominated, with fewer mixed diabetes cohorts and one type 1 study Table 1. In Ethiopia, several studies report prevalence levels between 61.1% and 73.8%. For instance, a meta‐analysis from the Oromia region documented a pooled prevalence of 61.1%, with higher rates observed among older adults and individuals with comorbidities [17]. Another study at Tikur Anbessa Specialized Hospital in Addis Ababa found a striking 73.8% of participants with poor glycemic control based on HbA1c [11]. Similarly, studies from Jimma and Adama reported prevalence rates of 70.9% and 64.1%, respectively [12, 18]. Hospital‐based studies indicated that glycemic control was significantly worse among older adults (≥ 50 years), individuals with no formal education, low income, or those engaged in farming. Clinically, poor control was associated with longer duration of diabetes, insulin‐based or combination therapy, poor medication or dietary adherence and lifestyle factors such as alcohol intake, khat chewing and physical inactivity.

**TABLE 1:** Epidemiological characteristics and related risk factors associated with the prevalence of poor glycemic control in the East Africa region.

Country	Study settings	Type of study	Marker of glycaemic control	Type of diabetes	Sample size	Prevalence of poor glycemic control	Demographic factors	Clinical/Lifestyle factors	References
Ethiopia	Study in Oromia, West Shewa	Facility‐based cross‐sectional study	HbA1c (poor control defined as average HbA1c ≥ 7%)	Diabetes mellitus (adult outpatients; DM type not specified)	390	63.80%	Age ≥ 50 years, female, single	High LDL‐C, alcohol intake, diabetic peripheral neuropathy.	Abdissa & Hirpa, [23]
Ethiopia	Tikur Anbessa Specialized Hospital, Addis Ababa	Hospital‐based cross‐sectional study	HbA1c (good < 7%; inadequate 7%–8%; poor > 8%; inadequate/poor HbA1c ≥ 7%)	Type 2 diabetes (T2DM)	325	73.8%	Older age (≥ 55 years)	Insulin therapy, poor diet adherence, failure to set glycemic targets.	Abera et al. [11]
Ethiopia	Jimma University Teaching Hospital	Facility‐based cross‐sectional survey	Fasting blood glucose (poor control FBG > 130 mg/dL)	Type 2 diabetes (T2DM)	325; 309 included in analysis	70.90%	Illiteracy, being a farmer	Combination insulin+oral therapy, poor medication adherence	Kassahun et al. [12]
Ethiopia	Oromia meta‐analysis (pooled)	Meta‐analysis	Poor glycaemic control as defined in included studies (HbA1c and/or fasting blood glucose, depending on study)	Type 2 diabetes (T2DM)	23 included studies; total participants 6643	61.1%	Older age > 50 years, no formal education	Long duration of DM (> 10 years), comorbidity, low adherence.	Tegegne et al. [17]
Ethiopia	Adama Hospital, East Ethiopia	Cross‐sectional study	Fasting blood glucose (mean of last three visits; poor control if outside 70–130 mg/dL range)	Type 2 diabetes (T2DM)	245	64.10%	Male gender, no formal education, low income	Overweight/obesity, khat chewing, lack of physical exercise.	Yosef et al. [18]
Uganda	Mbarara Regional Referral Hospital	Cross‐sectional study	HbA1c (poor control HbA1c ≥ 7%)	Type 2 diabetes (T2DM)	223	84.3% (HbA1c ≥ 7%)	Age 25–60 years and > 60 years	Non‐adherence to diet and physical exercise.	Patrick et al. [13]
Kenya	Kapkatet Sub‐county Hospital	Hospital‐based cross‐sectional study	Fasting blood glucose (poor control FBG ≥ 7.2 mmol/L)	Diabetes mellitus (adult patients; type not specified)	250 respondents (of 300 approached; 83.3% response)	60% (FBG ≥ 7.2 mmol/L)	NR	Duration > 10 years, insulin monotherapy, poor medication access.	Jemeli Rutto et al. [16]
Kenya	Kapkatet Sub‐county Hospital (South Rift) lifestyle study	Descriptive cross‐sectional study	HbA1c (most recent; good control HbA1c ≤ 7%)	Type 2 diabetes (T2DM)	300	61.2% (HbA1c > 7%)	NR	Poor physical activity, poor dietary adherence, poor medication adherence.	Ng'ambwa et al. [24]
Tanzania	Bugando Medical Centre, Mwanza (cross‐sectional)	Cross‐sectional study (plus secondary record review for complications)	HbA1c and random blood glucose (RBG); poor control HbA1c > 7% or RBG > 7 mmol/L	Type 1 and Type 2 diabetes	340 (primary sample); secondary data 7952 for complications	66.4%	Age 40–60 years, illiterate or only informal education	Long duration of DM (> 10 years), inadequate physical exercise, smoking.	Muchunguzi et al. [14]
Tanzania	Type 1 DM children/adolescents (HbA1c > 7.5%)	Cross‐sectional study	HbA1c (poor control HbA1c > 7.5%)	Type 1 diabetes (T1DM)	150	12.3% HbA1c 146	NR	BMI category, insulin type, caretaker education level.	McLarty et al. [25]
Rwanda	Kirehe District	Cross‐sectional study	HbA1c (uncontrolled HbA1c ≥ 7%; measured for the sample)	Type 2 diabetes (T2DM)	201	72.6%	Age, sex, education level, occupation, income, distance to health facility	Medication type, treatment length, comorbidities, physical exercise, compliance.	Dusabeyezu et al. [26]
Rwanda	Retrospective study at CHUK	Cross‐sectional study with retrospective chart review (facility‐based)	HbA1c (poorly controlled HbA1c at time of complication)	Diabetes mellitus (T1D and T2D; majority T2D among recorded types)	246 charts reviewed	95.2%	Age ≥ 45 years, T1DM type	Alcohol intake, longer duration of DM.	Iradukunda et al. [15]
Tanzania	Five hospitals with paediatric diabetes clinics (Kilimanjaro Christian Medical Centre; Muhimbili National Hospital; Sekou Toure Hospital; Mnazi Mmoja Hospital; Temeke Hospital)	Retrospective record review (2010–2016)	HbA1c (categories used: < 7.5% good; 7.5%–9.9% moderate; 10%–12.5% poor; > 12.5% very poor); fasting plasma glucose	Type 1 diabetes (T1D) (children/young adults followed in paediatric diabetes clinics)	559 included (of 604 screened)	Not reported	Descriptive only (age groups; sex ratio~1.1:1).	Contextual factors discussed: limited insulin supply and self‐monitoring resources; missing HbA1c/clinical documentation; poor monitoring practices. Complication risk related to older age at diagnosis for neuropathy (reported in survival analyses).	Najem et al. [27]
Rwanda	Primary‐level outpatient NCD clinics at health centers in Rwamagana District, plus Rwamagana Provincial Hospital follow‐up (rural/low‐resource primary care context)	Prospective cohort study (6‐month follow‐up)	HbA1c (controlled defined as HbA1c < 7%; poor control defined as HbA1c ≥ 7%)	Type 2 diabetes (T2DM)	130 enrolled at baseline; 123 followed at 6 months (*N* = 124 in baseline demographics table)	Poor control: 74% at baseline (HbA1c ≥ 7%); 63% at 6 months (HbA1c ≥ 7%)	Older age associated with achieving HbA1c < 7% after 6 months in univariable models; socioeconomic category (ubudehe) considered in models.	Higher baseline HbA1c strongly predicted larger HbA1c reductions but lower odds of achieving HbA1c < 7% after 6 months; BMI/overweight–obesity included; home‐based care practitioner exposure not associated with improved HbA1c outcomes.	Bahizi et al. [28]
Tanzania	Outpatient diabetes clinics at three facilities in Mwanza region: Bugando Medical Center (tertiary), Sekou Toure Hospital (regional) and Sengerema Hospital (district)	Cross‐sectional study	HbA1c measured (classification: ≤ 7.5% good; 7.5%–10% moderate; 10%–12.5% poor; ≥ 12.5% very poor); blood glucose also measured	Diabetes mellitus in children/adolescents (insulin‐dose history collected; diabetes type not explicitly stated in the article text)	155 children/adolescents (ages 5–19 years)	69% had poor glycaemic control (HbA1c > 10%)	Older age (13–19 years) associated with microvascular complications (and aligned with poorer control categories reported).	Longer duration of diabetes (> 5 years) and very poor glycaemic control were key risk factors for microvascular complications; insulin dosing/storage behaviours were captured as part of clinical history.	Msanga et al. [29]

In Uganda, Patrick et al. [13] reported a prevalence of 84.3% among T2D patients at Mbarara Regional Referral Hospital, highlighting one of the highest rates in the region. The middle‐aged and elderly adults are disproportionately affected, while non‐adherence to diet and physical inactivity were the main contributing factors. In Kenya, studies conducted in Kapkatet Sub‐County Hospital found glycemic control to be poor in 60% to 61.2% of patients, depending on the metrics used [16, 24]. Although the demographic data were not consistently reported, clinical correlates of poor control were long‐standing diabetes, insulin monotherapy and poor access to medications. The significant suboptimal lifestyle behaviours attributed were insufficient physical activity and dietary non‐adherence.

From Tanzania, a cross‐sectional study at Bugando Medical Centre reported a poor glycemic control prevalence of 66.4% [14], while paediatric populations in the same country showed mean HbA1c levels as high as 12.3%, indicating chronic poor control [27]. However, poor glycemic control was notably higher among individuals aged 40–60 years, those with informal education and those with long‐standing disease, smoking history, or low exercise levels. Although one study in Kirehe District did not specify prevalence numerically in Rwanda, high rates of uncontrolled diabetes were implied through clinical thresholds and patient profiles [26, 28]. And the highlighted influencing factors were education, occupation, and distance to health facilities. Other findings from Rwanda showed extremely high rates of poor control, with one retrospective study reporting 95.2% prevalence [15]. Contributing factors included older age (≥ 45 years), TID, alcohol use and longer disease duration.

Our findings, indicate that poor glycemic control varies across the region, highlighting systemic gaps in diabetes care and long‐term management. Generally, the prevalence of poor glycemic control in East African diabetes patients—ranging from 60% to over 84%—far exceeds global averages. However, a systematic review and meta‐analysis from sub‐Saharan Africa showed that glycaemic control prevalence ranged from 10%–60%, compared with 40%–55% in Europe and the U.S. [30]. Moreover, globally, only about 24% of insulin‐treated T2D patients meet glycemic targets [31]. These disparities underscore systemic deficiencies in diabetes management across East Africa, including limited access to medications, education and self‐management resources. Urgent policies are needed to strengthen primary care, enhance patient support structures and equitably improve glycemic outcomes in this high‐burden region.

### 3.2 Factors Associated With Poor Glycemic Control Among Diabetes Patients in the East African Region

The regional evidence synthesis of studies identifies various demographic, behavioural and clinical factors consistently associated with poor glycemic control across East Africa Table 1.

Demographic determinants include advanced age, low education levels and socioeconomic disadvantage. Studies from Ethiopia repeatedly show higher odds of poor glycemic control among patients aged 50 and those with no formal education [17, 18]. Similar findings were observed in Tanzania, where patients aged 40–60 and those with only informal education were more likely to experience poor control [14]. In Kenya, lack of education and low income were also reported among key contributors [16]. Further, individuals with lower socioeconomic status often experience higher rates of poor glycemic control due to barriers in education, resources and healthcare access [9, 13].

Clinical factors significantly influencing glycemic control include long duration of diabetes, insulin use, comorbid conditions and diabetic complications. The presence of comorbidities, such as hypertension and dyslipidemia, adversely affects glycemic control [32]. Patients with multiple health conditions may face additional treatment complexities, leading to suboptimal diabetes management. Comorbid conditions can exacerbate insulin resistance and create a cycle of poor metabolic control [33]. In Kenya and Ethiopia, studies found that patients living with diabetes for more than 10 years, or using insulin therapy alone, had poorer glycemic outcomes [12, 16]. Diabetic neuropathy and high LDL cholesterol were also independently associated with poor control in West Shewa, Ethiopia [23]. Also, psychosocial factors, mental health issues, such as depression and anxiety, can adversely affect diabetes management. Indicating that social support and educational resources can influence a patient's ability to maintain glycemic control [34]. The length of time since diabetes diagnosis is correlated with glycemic control; longer disease duration often leads to progressive *β*‐cell dysfunction and increased insulin resistance, making glycemic control more challenging [35]. Research indicates that patients diagnosed for a longer time may experience worsening HbA1c levels due to complications and treatment fatigue [36].

Behavioural and lifestyle‐related variables such as physical inactivity, poor dietary adherence and substance use (e.g., alcohol or khat chewing) are consistently reported across multiple countries in the region. Lifestyle factors, diet, physical activity and sedentary behaviour are critical in managing blood glucose levels [37]. Our evidence synthesis shows that in Uganda, non‐adherence to dietary recommendations and lack of physical activity were key predictors of poor control [13]. Similarly, poor medication and diet adherence in Kenya strongly correlated with elevated blood glucose levels [24]. Additionally, systemic factors such as poor access to medication, absence of self‐monitoring tools and limited diabetes education exacerbate the situation. However, none of the included studies clearly reported the level of access to medicines for glycaemic control, including whether treatment was provided free of charge, paid for out‐of‐pocket, or variably covered across settings; this limits interpretation of medication non‐adherence and its policy implications. In Rwanda, variables such as treatment duration, distance to health facilities and patient compliance significantly influenced glycemic outcomes [26]. Non‐adherence to treatment or poor adherence to prescribed medication regimens can significantly impact glycemic control. Patients may forget doses, misunderstand instructions, or intentionally skip medications due to side effects or lack of perceived benefit [38]. Studies indicate that factors contributing to non‐adherence include complexity of medication regimens, side effects and lack of understanding of the treatment importance [39]. Effective communication between healthcare providers and patients is essential for successful diabetes management. Education regarding self‐monitoring of blood glucose, lifestyle modifications and medication adherence improves patient outcomes [40]. The quality of the healthcare provider‐patient relationship can directly influence patients' motivation and capability to manage their diabetes [40]. Limited access to healthcare services among the resource‐limited region of the East African community significantly impacts glycemic control, inadequate access to glucose monitoring tools, medications and regular consultations leading to poor management of diabetes [41]. Other studies have shown that patients with restricted access are less likely to adhere to treatment regimens, resulting in higher HbA1c levels and increased risk of complications [9, 41]. Collectively, these findings emphasize the multifactorial nature of glycemic control and underscore the need for integrated and patient‐centred diabetes management strategies.

### 3.3 Knowledge Gaps and Limitations of East African Epidemiological Prevalence of Poor Glycemic Control

Despite a growing body of research on glycemic control in East Africa, significant knowledge gaps and methodological limitations persist across studies conducted in Ethiopia, Kenya, Uganda, Tanzania and Rwanda Table 2. Most studies employ cross‐sectional designs [13, 28, 42] which, although useful for identifying associations, limit the ability to draw causal inferences about factors affecting glycemic control. Furthermore, many of these studies are hospital‐based, often conducted in urban tertiary care centres, lacking representation of rural populations or patients managed in primary care settings [28]. This raises concerns about external validity and generalizability to broader diabetic populations. Many studies on glycemic control utilize cross‐sectional designs, which capture data at a single point in time. This approach limits the ability to establish causal relationships between various risk factors (like diet, exercise and medication adherence) and poor glycemic control [43]. Without longitudinal data, it's challenging to determine whether these factors precede or result from poor control [32, 44]. Another recurrent limitation is the underreporting or inconsistent measurement of behavioural and psychosocial variables such as dietary adherence, self‐monitoring practices and diabetes education—factors that are crucial to understanding glycemic outcomes. Additionally, retrospective designs, particularly in paediatric populations [27], often suffer from incomplete or missing records, reducing the reliability of findings.

**TABLE 2:** Scientific findings, methods, limitations, gaps and future focus on the prevalence of poor glycaemic control in the East Africa region.

References	Country	Study design	Methods	Findings	Limitations	Research gaps	Future focus
Muchunguzi et al. [14]	Tanzania	Bugando Medical Centre (cross‐sectional)	Facility‐based survey, logistic regression	Longer diabetes duration, insulin use, no glycemic targets ↑ odds of poor control	Not generalizable nationally; cross‐sectional design limits causality	Regional scope; lack of longitudinal data	Prospective cohort studies, interventions to set glycemic targets
Abera et al. [11]	Ethiopia	Tikur Anbessa Specialized Hospital (cross‐sectional with NGSP‐certified HbA1c)	HbA1c measurement (NGSP/DCCT‐standard), diet adherence, self‐monitoring assessed	73.8% poor control; diet non‐adherence and lack of target goals significant	Likely skewed towards more complex referred cases; urban, selective sample	Rural and community‐level data lacking	Broadened sampling, device access studies, patient‐targeted programmes
Najem et al. [27]	Tanzania	Type 1 DM paediatric retrospective	Chart review of children/adolescents (HbA1c & complications)	36% had HbA1c > 12.5%; high retinopathy (21.5%) and neuropathy (29.4%) despite short duration	Missing data; retrospective nature; no nephropathy info; caution needed	Poor record‐keeping; prospective validation needed	Prospective paediatric cohort studies with complete records
Msanga et al. [29]	Tanzania	Cross‐sectional paediatric DM complications	Interviews, clinical exams, lab tests on children/adolescents	Detailed clinical data collection; used monofilament, fundoscopy, HbA1c categorization	Cross‐sectional, limited generalizability	Intervention trials lacking	Implement prevention interventions, longitudinal complication tracking
Bahizi et al. [28]	Rwanda	Prospective cohort in primary care	Structured interviews and HbA1c at baseline and 6 months; regression analysis	HbA1c < 7% improved from 26% to 37%; baseline HbA1c > 9% predicted better improvement; home‐based care not effective	Short follow‐up; small sample; limited generalizability	Long‐term outcomes and broader decentralized care effects	Longer‐term monitoring, RCTs of community care interventions
Dusabeyezu et al. [26]	Rwanda	Kirehe district cross‐sectional	Survey plus multivariable analysis	Identified demographic and clinical predictors of uncontrolled DM; exact prevalence unspecified	No prevalence reported; district‐level scope	Prevalence data; generalizable metrics	Full prevalence studies; multi‐site rural–urban comparisons
Patrick et al. [13]	Uganda	Mbarara Regional Referral Hospital (cross‐sectional)	Clinic‐based survey (details from literature)	High poor control; behavioural contributors like poor diet and exercise	Likely single‐centre bias; design limits causality	Objective behaviour tracking; broader demographic sampling	Behaviour‐change intervention studies; multicenter research

In Rwanda and Tanzania, studies showed insufficient tracking of long‐term outcomes and inadequate evaluation of community‐based interventions [26, 28]. For instance, home‐based care in Rwanda did not demonstrate significant impact, yet its underlying components and implementation fidelity remain unclear. There is also a lack of consistent use of standardized glycemic control metrics (e.g., HbA1c), particularly in resource‐limited settings where biochemical testing is not routinely available [42]. Variability in HbA1c cutoffs different studies use varying thresholds for defining poor glycemic control, leading to difficulties in comparing results, for instance, some studies consider an HbA1c level of > 7% (53 mmol/mol) as the cutoff, while others may use > 6.5% (48 mmol/mol) or > 8% (64 mmol/mol) [45]. This inconsistency can distort the prevalence estimates and associated risk factors for poor glycemic control [44, 46]. There is a notable absence of standardized treatment protocols in the region, and there are various geographic and demographic factors, such as differences in medication availability, healthcare practices and patient education [47]. Also, comparative effectiveness and lack of uniformity in interventions complicate this assessment because what works in one population or healthcare setting may not be applicable in another, making it difficult to generalize findings in the East Africa region [48].

## 4 Discussion

The included studies consistently indicate that poor glycaemic control is highly prevalent in East Africa, but interpretation must be framed by constraints in measurement, that is, comparisons require caution because markers and thresholds varied across settings (limited HbA1c access in many settings), selection bias (predominantly facility‐based samples) and heterogeneous thresholds across studies (Abdissa and Hirpa, [23]; Kassahun et al. [12]; Muchunguzi et al. [14]). Facility‐based cross‐sectional designs may over‐represent more severe or poorly served patients, potentially inflating prevalence estimates (Patrick et al. [13]; Abera et al. [11]). The meta‐analysis confirms the overall burden while highlighting substantial heterogeneity (Tegegne et al. [17]). More standardized HbA1c reporting, consistent thresholds and clearer diabetes‐type classification would improve interpretability and better guide context‐appropriate interventions. Routine availability of HbA1c testing and adherence support remain critical priorities. The most repeatable correlates were duration of diabetes, treatment intensity (often insulin use as a marker of advanced disease), comorbidities/complications and modifiable adherence‐ and access‐related factors.

### 4.1 Integrated Strategies and Approaches for Improving Glycemic Control and Future Research Directions

Effective diabetes management requires a patient‐centred approach that aligns treatment strategies with individual values, beliefs and daily routines. Involving patients in shared decision‐making improves adherence and satisfaction [49]. Lifestyle interventions should consider dietary patterns, physical activity and occupational demands, while addressing psychosocial factors—such as stress, emotional health and social support—which are essential for sustainable behaviour change [50]. Motivational interviewing is valuable for exploring barriers and fostering self‐efficacy [1].

Technology and therapeutics—contextual feasibility: Where available, HbA1c testing and structured self‐monitoring can improve titration and adherence, but many East African settings face intermittent access to strips, insulin, oral agents and laboratory assays. Therefore, proposed innovations should be tiered: (i) minimum package (reliable medicine supply, BP/lipid management, education, simplified regimens), (ii) enhanced package (periodic HbA1c, SMBG for insulin users, task‐shared follow‐up) and (iii) advanced package (CGM/pump therapy for selected patients) only where financing, supply chains and clinical support exist. Newer agents (e.g., GLP‐1RA/SGLT2 inhibitors or dual agonists) are discussed as future options contingent on affordability and national formularies, rather than near‐term solutions across the region. To bridge the existing gaps and enhance diabetes management in East Africa, future research must prioritize longitudinal and interventional study designs. Prospective cohort studies can provide valuable insights into the progression of glycemic control over time and identify temporal relationships between clinical, behavioural and system‐level factors [26, 28]. These designs are especially needed in paediatric and adolescent populations with T1D, where early‐onset complications are prevalent but understudied [29].

Additionally, future research should expand beyond urban tertiary centres to include rural and decentralized primary care settings. These environments represent a significant proportion of the diabetic population but are often excluded from major studies. Comparative analyses between rural and urban patients could elucidate context‐specific barriers such as healthcare access, medication availability and sociocultural beliefs [13, 26].

Behavioural interventions also warrant further exploration. Few studies have rigorously tested the effectiveness of dietary counselling, self‐monitoring support, or community health worker–led programmes in improving glycemic control. Given the high prevalence of poor adherence reported across multiple studies, randomized controlled trials (RCTs) targeting behavioural change and patient education could be impactful [42].

Moreover, digital health technologies—including mobile‐based glucose monitoring and telemedicine—should be evaluated for feasibility and impact in low‐resource settings. Rwanda's limited success with home‐based care underscores the need for clearer implementation frameworks and monitoring tools to assess intervention fidelity and outcomes [28]. Lastly, research should prioritize developing and validating culturally appropriate diabetes education tools and risk assessment models tailored to East African populations. Strengthening the evidence base in these areas will enhance glycemic control and inform national diabetes strategies and health system planning.

## 5 Conclusion

This review highlights a persistent prevalence of poor glycemic control among people living with diabetes mellitus across the East Africa region. The poor glycemic outcomes are consistently associated with sociodemographic variables such as older age and low education. Also, poor glycemic conditions are impacted by clinical factors including longer disease duration, insulin‐only therapy and patient behavioural challenges such as poor adherence to diet and medication; the lack of self‐monitoring is a contributing factor associated with worse glycaemic outcomes. To completely comprehend the dynamics of glycaemic management in various countries, longitudinal, community‐level research is essential, as evidenced by the preponderance of cross‐sectional, facility‐based studies. A multi‐sectoral strategy that includes integrating behavioural and culturally specific therapies, decentralizing services, and improving diabetes education is needed in the region. Strengthening primary care systems and promoting evidence‐based and patient‐centred management are essential steps towards improving glycaemic outcomes and lowering diabetes‐related morbidity in the region.

## Author Contributions

**Fanny Eseohe Onohuean:** conceptualization, methodology, validation, investigation, data curation, formal analysis, writing – original draft. **Haron Olot:** conceptualization, investigation, writing – original draft, methodology, validation, data curation. **Hope Onohuean:** conceptualization, investigation, writing – original draft, methodology, validation, writing – review and editing, visualization, project administration, formal analysis, software, supervision, resources, data curation. **Mary Onohuean:** investigation, methodology, validation, formal analysis, data curation, writing – original draft.

## Funding

The authors have nothing to report.

## Consent

All the authors have read and agreed to the final copy of the manuscript.

## Conflicts of Interest

The authors declare no conflicts of interest.

## Supporting information


**Data S1:** Detailed search strategies used across PubMed, Scopus and Web of Science, the corresponding search terms and retrieval dates, database‐specific record counts and the study selection workflow. It also contains the PRISMA flow diagram summarizing identification, screening, eligibility assessment, duplicate removal and final study inclusion for the meta‐synthesis and review.

## Data Availability

The data that supports the findings of this study are available in the supporting information (Data S1) of this article.
