# Supplementary material for: Poor Glycemic Control in East Africa: Prevalence, Risk Factors and Public Health Implications in Diabetes Management
Source: Endocrinol Diabetes Metab. 2026 May 4;9(3):e70233. doi: 10.1002/edm2.70233 (PMC13139639; doi:10.1002/edm2.70233)
Supplement: Supplementary file 1 — Data S1: Detailed search strategies used across PubMed, Scopus and Web of Science, the corresponding search terms and retrieval dates, database‐specific record counts and the study selection workflow. It also contains the PRISMA flow diagram summarizing identification, screening, eligibility assessment, duplicate removal and final study inclusion for the meta‐synthesis and review. [file EDM2-9-e70233-s001.docx]

**Poor Glycemic Control in East Africa: Prevalence, Risk Factors, and Public Health Implications in Diabetes Management**

Fanny Eseohe Onohuean^1^, Mary Onohuean^1^, Haron Olot^2,3^, Hope Onohuean^*,2,3^

^1^ Department of Nursing and Midwifery, Faculty of Health Science, Metropolitan International University, Kisoro District, Uganda

^2^ Biomolecules, Metagenomics, Endocrine & Tropical Disease Research Group (BMETDREG), Kampala International University, Western Campus, Ishaka-Bushenyi, Uganda

^3^Biopharmaceutics unit, Department of Pharmacology & Toxicology, School of Pharmacy, Kampala International University, Western-Campus, Ishaka-Bushenyi, Uganda

*Author for correspondence: onohuean@gmail.com

Supplementary files

**Study selection**

Keywords: "diabetes mellitus," "glycaemic control," "HbA1c," "prevalence," "risk factors," and "East Africa,"

PubMed 05/09/2025.

Searched terms; ("Diabetes Mellitus"[MeSH Terms] OR "diabetes mellitus"[All Fields]) AND ("Glycemic Control"[MeSH Terms] OR "glycaemic control"[All Fields] OR "glycemic control"[All Fields]) AND ("Hemoglobin A, Glycosylated"[MeSH Terms] OR "HbA1c"[All Fields]) AND ("Prevalence"[MeSH Terms] OR "prevalence"[All Fields]) AND ("Risk Factors"[MeSH Terms] OR "risk factors"[All Fields]) AND (Kenya[All Fields] OR Ethiopia[All Fields] OR Uganda[All Fields] OR Tanzania[All Fields] OR Rwanda[All Fields])

<https://pubmed.ncbi.nlm.nih.gov/?term=%28%22Diabetes+Mellitus%22%5BMeSH+Terms%5D+OR+%22diabetes+mellitus%22%5BAll+Fields%5D%29+AND+%28%22Glycemic+Control%22%5BMeSH+Terms%5D+OR+%22glycaemic+control%22%5BAll+Fields%5D+OR+%22glycemic+control%22%5BAll+Fields%5D%29+AND+%28%22Hemoglobin+A%2C+Glycosylated%22%5BMeSH+Terms%5D+OR+%22HbA1c%22%5BAll+Fields%5D%29+AND+%28%22Prevalence%22%5BMeSH+Terms%5D+OR+%22prevalence%22%5BAll+Fields%5D%29+AND+%28%22Risk+Factors%22%5BMeSH+Terms%5D+OR+%22risk+factors%22%5BAll+Fields%5D%29+AND+%28Kenya%5BAll+Fields%5D+OR+Ethiopia%5BAll+Fields%5D+OR+Uganda%5BAll+Fields%5D+OR+Tanzania%5BAll+Fields%5D+OR+Rwanda%5BAll+Fields%5D%29&filter=years.2015-2025&size=100>

10 results

Scopus

Search within: Article title, Abstracts, Keywords, Authors:

TITLE-ABS-KEY: ( diabetes mellitus ) AND ( glycaemic control OR glycemic control ) AND ( HbA1c OR glycated hemoglobin OR glycosylated hemoglobin ) AND ( prevalence ) AND ( risk factors ) AND ( Ethiopia OR Kenya OR Uganda OR Tanzania OR Rwanda )

2002 to 2025 Results: 23 documents found

Limiting to 2015 to 2025 Results: 19 documents found.

<https://0-www-scopus-com.innopac.wits.ac.za/results/results.uri?st1=%28diabetes+mellitus%29+AND+%28glycaemic+control+OR+glycemic+control%29+AND+%28HbA1c+OR+glycated+hemoglobin+OR+glycosylated+hemoglobin%29+AND+%28prevalence%29+AND+%28risk+factors%29+AND+%28Ethiopia+OR+Kenya+OR+Uganda+OR+Tanzania+OR+Rwanda%29&st2=&s=TITLE-ABS-KEY%28%28diabetes+mellitus%29+AND+%28glycaemic+control+OR+glycemic+control%29+AND+%28HbA1c+OR+glycated+hemoglobin+OR+glycosylated+hemoglobin%29+AND+%28prevalence%29+AND+%28risk+factors%29+AND+%28Ethiopia+OR+Kenya+OR+Uganda+OR+Tanzania+OR+Rwanda%29%29&limit=10&origin=searchbasic&sort=plf-f&src=s&sot=b&sdt=b&sessionSearchId=ac3460be9b9ec9b2eb34d06848f7ba79>

WOS

Search within Topics: ( diabetes mellitus ) AND ( glycaemic control OR glycemic control ) AND ( HbA1c OR glycated hemoglobin OR glycosylated hemoglobin ) AND ( prevalence ) AND ( risk factors ) AND ( Ethiopia OR Kenya OR Uganda OR Tanzania OR Rwanda )

1997 to 2025 Results:17 results

Excluded Review articles 2, 1997 1.

Results: 14.

<https://0-www-webofscience-com.innopac.wits.ac.za/wos/woscc/summary/4fc1f43e-0555-4e64-8811-a42f2881f758-0177f46a17/129aeffb-dbb6-42f9-a35f-cd8813a724dd-0177f38243/relevance/1>
